# Benchmarking Spike-Based Visual Recognition: A Dataset and Evaluation

**DOI:** 10.3389/fnins.2016.00496

**Published:** 2016-11-02

**Authors:** Qian Liu, Garibaldi Pineda-García, Evangelos Stromatias, Teresa Serrano-Gotarredona, Steve B. Furber

**Affiliations:** ^1^Advanced Processor Technologies Research Group, School of Computer Science, University of ManchesterManchester, UK; ^2^Instituto de Microelectrónica de Sevilla (IMSE-CNM) - CSICSevilla, Spain

**Keywords:** benchmarking, vision dataset, evaluation, neuromorphic engineering, spiking neural networks

## Abstract

Today, increasing attention is being paid to research into spike-based neural computation both to gain a better understanding of the brain and to explore biologically-inspired computation. Within this field, the primate visual pathway and its hierarchical organization have been extensively studied. Spiking Neural Networks (SNNs), inspired by the understanding of observed biological structure and function, have been successfully applied to visual recognition and classification tasks. In addition, implementations on neuromorphic hardware have enabled large-scale networks to run in (or even faster than) real time, making spike-based neural vision processing accessible on mobile robots. Neuromorphic sensors such as silicon retinas are able to feed such mobile systems with real-time visual stimuli. A new set of vision benchmarks for spike-based neural processing are now needed to measure progress quantitatively within this rapidly advancing field. We propose that a large dataset of spike-based visual stimuli is needed to provide meaningful comparisons between different systems, and a corresponding evaluation methodology is also required to measure the performance of SNN models and their hardware implementations. In this paper we first propose an initial NE (Neuromorphic Engineering) dataset based on standard computer vision benchmarksand that uses digits from the MNIST database. This dataset is compatible with the state of current research on spike-based image recognition. The corresponding spike trains are produced using a range of techniques: rate-based Poisson spike generation, rank order encoding, and recorded output from a silicon retina with both flashing and oscillating input stimuli. In addition, a complementary evaluation methodology is presented to assess both model-level and hardware-level performance. Finally, we demonstrate the use of the dataset and the evaluation methodology using two SNN models to validate the performance of the models and their hardware implementations. With this dataset we hope to (1) promote meaningful comparison between algorithms in the field of neural computation, (2) allow comparison with conventional image recognition methods, (3) provide an assessment of the state of the art in spike-based visual recognition, and (4) help researchers identify future directions and advance the field.

## 1. Introduction

Researchers are using the capabilities created by rapid developments in neuromorphic engineering to address the dual aims of understanding brain functions and building brain-like machines (Furber and Temple, 2007). Neuromorphic engineering has delivered biologically-inspired sensors such as DVS (Dynamic Vision Sensor) silicon retinas (Delbruck, 2008; Serrano-Gotarredona and Linares-Barranco, 2013; Posch et al., 2014; Yang et al., 2015), which offer the prospect of low-cost visual processing thanks to their event-driven and redundancy-reducing style of information representation. Moreover, SNN simulation tools (Gewaltig and Diesmann, 2007; Davison et al., 2008; Goodman and Brette, 2008) and neuromorphic hardware platforms (Schemmel et al., 2010; Benjamin et al., 2014; Furber et al., 2014; Merolla et al., 2014) have been developed to allow exploration of the brain by mimicking its functions and developing large-scale practical applications (Eliasmith et al., 2012). Achieving the brain's energy efficiency motivates the development of neuromorphic hardware, since the human brain consumes only about 20 W of power (Drubach, 2000). In the case of visual processing, the brain can accurately recognize objects remarkably quickly, e.g., in 200 ms in monkeys (Fabre-Thorpe et al., 1998), even with short presentations (less than 100 ms) of the target objects (Keysers et al., 2001). Such rapid and highly accurate recognition is the target of modeling spike-based visual recognition.

Inspired by biological studies of the visual ventral pathway, SNN models have successfully been adapted to visual recognition. Riesenhuber and Poggio (1999) proposed a quantitative modeling framework for object recognition with position-, scale- and view-invariance. Their cortex-like model has been analyzed on several datasets (Serre et al., 2007). Recently Fu et al. (2012) reported that their SNN implementation was capable of recognizing facial expressions with a classification accuracy (CA) of 97.35% on the JAFFE dataset (Lyons et al., 1998) which contains 213 images of 7 facial expressions posed by 10 individuals. According to Van Rullen and Thorpe (2002), the first wave of spikes carry explicit information through the ventral stream and in each stage meaningful information is extracted and spikes are regenerated. Using one spike per neuron, similar to the first spiking wave in biology, Delorme and Thorpe (2001) reported 100% and 97.5% accuracies on the face identification task over training (40 individuals × 8 images) and testing data (40 individuals × 2 images). Hubel and Wiesel (1962) first discovered the model of orientation selectivity (simple cells) and pooling mechanism (complex cells) in the primary cortex in cats, which lay the foundation of the Convolutional Neural Network (CNN) (LeCun et al., 1998). An early Convolutional Spiking Neural Network (CSNN) model identified the faces of 35 persons with a CA of 98.3% exploiting simple integrate and fire neurons (Matsugu et al., 2002). Another CSNN model (Zhao et al., 2015) was trained and tested both with DVS raw data and Leaky Integrate-and-Fire (LIF) neurons. It was capable of recognizing three moving postures with a CA of about 99.48% and classifying hand-written digits with 88.14% accuracy on the MNIST-DVS dataset (see Section 2.2). In a further step forward, Camunas-Mesa et al. (2012) implemented a convolution processor module in hardware which could be combined with a DVS for high-speed recognition tasks. The inputs of the ConvNet were continuous spike events instead of static images or frame-based videos. The chip was capable of detecting the four suits in a 52-card deck which was browsed rapidly in only 410 ms. Similarly, a real-time gesture recognition model (Liu and Furber, 2015) was implemented on a neuromorphic system with a DVS as a front-end and a SpiNNaker (Furber et al., 2014) machine as the back-end, where LIF neurons built up the ConvNet configured with biological parameters. In this study's largest configuration, a network of 74,210 neurons and 15,216,512 synapses used 290 SpiNNaker cores in parallel and reached 93.0% accuracy. Spike-Timing-Dependent Plasticity (STDP) as a learning mechanism based on biological observations has been applied to vision tasks. Bichler et al. (2012) demonstrated an unsupervised STDP learning model to classify car trajectories captured with a DVS retina. A similar model was tested on a Poissonian spike presentation of the MNIST dataset achieving a performance of 95.0% (Diehl and Cook, 2015). Theoretical analysis (Nessler et al., 2013) showed that unsupervised STDP was able to approximate a stochastic version of Expectation Maximization, a powerful learning algorithm in machine learning. A computer simulation achieved a 93.3% CA on MNIST and had the potential to be implemented using memristors (Bill and Legenstein, 2014).

Deep Neural Networks (DNNs) have exceeded human-level performance on image classification tasks (He et al., 2015), but mainstream DNN research is focussed on continuous rather than spiking neural networks. The spiking deep network has great potential to combine remarkable performance with energy-efficient training and operation. Early research into spiking deep networks focussed on converting off-line trained deep network into SNNs (O'Connor et al., 2013). The network was initially implemented on an FPGA and achieved a CA of 92.0% (Neil and Liu, 2014), while a later implementation on SpiNNaker scored 95.0% (Stromatias et al., 2015b). Recent advances have contributed to better translation by using modified units in a ConvNet (Cao et al., 2015) and tuning the weights and thresholds (Diehl et al., 2015). The latter paper claims a state-of-the-art performance (99.1% on the MNIST dataset) compared to the original ConvNet. The current trend toward training Spiking DNNs on line using biologically-plausible learning methods is also promising. An event-driven Contrastive Divergence (CD) training algorithm for Restricted Boltzmann Machines (RBMs) was proposed for Deep Belief Networks (DBNs) using LIF neurons with STDP synapses and verified on MNIST with a CA of 91.9% (Neftci et al., 2013).

Despite the promising research on SNN-based vision recognition, there is no commonly-used database in the format of spike stimuli. In the studies listed above, all of the vision data used are in one of the following formats: (1) raw gray-scale images data; (2) pixel-intensity-driven rate-based Poisson spike trains; (3) unpublished spike-based videos recorded from DVS silicon retinas. However, in the field of conventional non-spiking computer vision, there are a number of datasets playing important roles at different times and with various objectives (LeCun et al., 1998; Blank et al., 2005; Deng et al., 2009; Liu et al., 2009). In consequence, a new set of spike-based vision datasets is now needed to quantitatively measure progress within the rapidly advancing field of spike-based visual recognition and to provide resources to support fair competition between researchers.

Apart from using spikes instead of the frame-based data used in conventional computer vision, new concerns arise when evaluating neuromorphic vision, such as latency and energy consumption, in addition to recognition accuracy. These concerns naturally derive from the goal of spike-based visual recognition: mimicking the fast recognition with low-energy processing in the brain. Therefore a set of common metrics for performance evaluation in spike-based vision is required to assess SNN models and their hardware implementations. In this paper we propose a large dataset of spike-based visual stimuli and a complementary evaluation methodology. Just as research in this field is an expanding and evolving activity, the dataset will be adapted and extended to fit new requirements presented by advances in the field.

The rest of this paper is structured as follows: Section 2 elaborates the purpose and protocols of the proposed dataset and describes the sub-datasets and the methods employed to generate them; it also demonstrates the suggested evaluation methodology for use with the dataset. Section 3 presents two SNNs as demonstrations of using the dataset to assess model performance and benchmark hardware platforms. Finally, Section 4 summarizes the paper and discusses future work.

## 2. Materials and methods

### 2.1. Guiding principles

The NE database we propose here is a developing and evolving dataset consisting of various spike-based representations of images and videos. The spikes are either generated from spike encoding methods which convert images or frames of videos into spike trains, or recorded from DVS silicon retinas. The spike trains are in the format of Address-Event Representation (AER) (Mahowald, 1992) data, which are suitable for both event-driven computer simulations and neuromorphic systems. AER was originally proposed as a time-multiplexed spike communication protocol where each time a neuron produces a spike an event is generated that codes the spiking neuron's address on a fast time-multiplexed digital bus. The recorded AER data consists of a list of events, each one containing the time stamp of a spike and the address of the neuron which generated the spike. With the NE dataset we hope:

- *to promote meaningful comparisons of algorithms in the field of spiking neural computation*. The NE dataset provides a unified format of AER data to meet the demands of spike-based visual stimuli. It also encourages researchers to publish and contribute their data to build up the NE dataset.
- *to allow comparison with conventional image recognition methods*. We expect the dataset to support this comparison using spiking versions of existing vision datasets. Thus, conversion methods are required to transform datasets of images and frame-based videos into spike stimuli. More biologically-accurate and better information preserving schemes are welcome.
- *to provide an assessment of the state of the art in spike-based visual recognition on neuromorphic hardware*. To reveal the accuracy, speed, and energy-efficient recognition of neuromorphic approaches, we need not only a spike-based dataset but also an appropriate evaluation methodology. The evaluation methodology will be constantly improving along with the evolution of the dataset.
- *to help researchers identify future directions and advance the field*. The development of the dataset and its evaluation methodology will introduce new challenges for the neuromorphic engineering community. However, these must represent an appropriate degree of difficulty: a too-easily-solved problem turns into a tuning competition, while a problem that is too difficult will not yield meaningful assessment. So suitable problems should continuously be added to promote future research.

### 2.2. The dataset: NE15-MNIST

The first proposed dataset in the benchmarking system is NE15-MNIST (Neuromorphic Engineering 2015 on MNIST). NE15-MNIST is the spiking version of an original non-spiking dataset which was downloaded from the MNIST Database of Handwritten Digits (LeCun et al., 1998) website^1^. Due to its straightforward target of classifying real-world images, the plain format of the binary data and simple patterns, MNIST has been one of the most popular datasets in computer vision for over 20 years. MNIST is a popular task among the neuromorphic vision research community as stated in Section 1. The converted MNIST dataset consists of four subsets which were generated for different purposes:

- *Poissonian*, which encodes each pixel as a Poisson spike train and is intended for benchmarking existing rate-based SNN models.
- *FoCal (Filter Overlap Correction ALgorithm)*, to promote the study of spatio-temporal algorithms applied to recognition tasks using small numbers of input spikes.
- *DVS recorded flashing input*, to encourage research into fast recognition methods to mimic the rapid and accurate “core recognition” in the primate ventral visual pathway (DiCarlo et al., 2012).
- *DVS recorded moving input*, to trigger the study of algorithms targeting continuous input from real-world sensors for implementation, for example, on mobile neuromorphic robots.

The dataset can be found in the GitHub repository at: https://github.com/NEvision/NE15.

### 2.3. Data description

**Figure 1:**
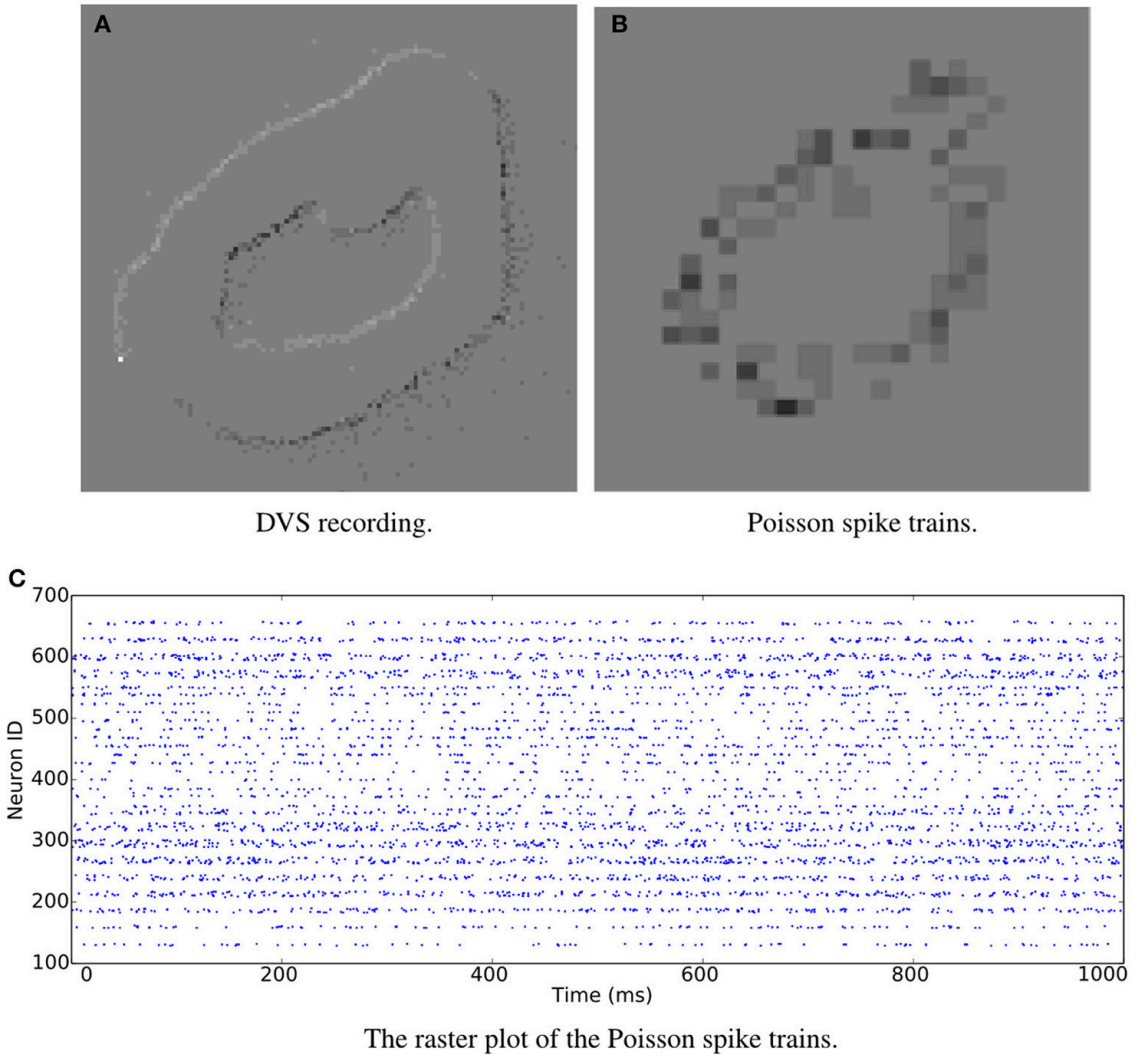
**Snapshots of the jAER software displaying spike-encoded videos**. The same image of digit “0” is transformed into spikes by **(A)** DVS recording and **(B)** Poisson generation. **(C)** A raster plot of the Poisson spike trains.

Two file formats are supported in the dataset: the jAER format (Delbruck, 2008) (.dat or .aedat), and binary files in NumPy (van der Walt et al., 2011) (.npy) format. The spikes in jAER format, whether recorded from a DVS retina or artificially generated, can be displayed by the jAER software. Figure 1A is a snapshot of the software displaying a .aedat file which was recorded from a DVS retina (Serrano-Gotarredona and Linares-Barranco, 2013). The resolution of the DVS recorded data is 128 × 128. The second spike-based format used is a list of spike source arrays in PyNN (Davison et al., 2008), a description language for building spiking neuronal network models. Python code is provided for converting from either file format to the other. The duration of the artificially-generated data can be configured using the Python code provided, while the recorded data varies in duration: 1 s for the flashing input, and 3.2–3.4 s for the moving input.

#### 2.3.1. Poissonian

The timing of spikes in the cortex is highly irregular (Squire and Kosslyn, 1998). An interpretation is that the inter-spike interval reflects a random process driven by the instantaneous firing rate. If the generation of each spike is assumed to be independent of all other spikes, the spike train is seen as a Poisson process. The spike rate can be estimated by averaging the pooled responses of the neurons.

As stated above, rate coding is generally used in presenting images as spike trains. The spike rate of each neuron accords with the intensity of the corresponding pixel. Instead of providing exact spike arrays, we share the Python code for generating the spikes. Each recognition system may require different spike rates and durations. The generated Poisson spike trains can be in both jAER and PyNN spike source array formats. Thus, it is easy to visualize the digits and also to couple the spike trains into spiking neural networks. Because different simulators generate random Poisson spike trains with different mechanisms, languages and codes, using the same dataset enables performance evaluation on different simulators without the confusion created by differences in input. The same digit displayed in Figure 1A can be converted into Poisson spike trains, see Figure 1B. A raster plot of the Poisson spike trains is shown in Figure 1C.

#### 2.3.2. Rank order encoding

A different way of encoding spikes is to use a rank order code; this means keeping just the order in which the spikes fired and disregarding their exact timing. Rank-ordered spike trains have been used in vision tasks under a biological plausibility constraint, making them a viable way of encoding images for neural applications (Van Rullen and Thorpe, 2001; Sen and Furber, 2009; Masmoudi et al., 2010).

Rank order coding (ROC) can be performed using an algorithm known as the FoCal algorithm (Sen and Furber, 2009). This algorithm models the foveola, the highest resolution area of the retina, with four ganglion cell layers each with a different scale of center-surround receptive field (Kolb, 2003). To simulate these layers two steps are required: the first consists of four discrete 2D convolutions; the second removes redundant information produced in the first step. During the first step, the center-surround behavior of the ganglion cells is modeled using Difference of Gaussians (DoG) kernel for convolution.

(1) $$\begin{array}{l} DoG_{w}\left(x,y\right)=\pm \frac{1}{2\pi \sigma_{w,c}^{2}}e^{\frac{-\left(x^{2}+y^{2}\right)}{2\sigma_{w,c}^{2}}}\mp \frac{1}{2\pi \sigma_{w,s}^{2}}e^{\frac{-\left(x^{2}+y^{2}\right)}{2\sigma_{w,s}^{2}}} \end{array}$$

where σ_*w, c*_ and σ_*w, s*_ are the standard deviation of the center and surround components of the DoG at layer *w*. The signs will be (−,+) if the ganglion cell has an OFF-center behavior and (+,−) if it has an ON-center one. Supplementary Table 1 shows the parameters (described in Sen and Furber, 2009) used to compute the convolution kernels at each scale *w*.

Every pixel value in the convolved image (Supplementary Figure 1) is inversely proportional to the spike emission time relative to the presentation of the image (i.e., the higher the pixel value, the sooner the spike will fire.)

Since DoGs are used as the means to encode the image, and they do not form an orthogonal set of basis functions, the algorithm also performs a redundancy correction step. It does so by adjusting the convolved images' pixel values according to the correlation between convolution kernels (Algorithm 1).

**Table:** **Algorithm 1** FoCal, redundancy correction

**procedure** Correction(coeffs *C*, correlations *Q*)
*N* ← ∅ ⊳ Corrected coefficients
**repeat**
*m* ← *max*(*C*) ⊳ Obtain maximum from *C*
*M* ← *M*∪*m* ⊳ Add maximum to *M*
*C* ← *C* \ *m* ⊳ Remove maximum from *C*
**for all** *c* ∈ *C* **do** ⊳ Adjust all remaining *c*
**if** *Q*(*m, c*) ≠ 0 **then** ⊳ Adjust only spatially near coefficients
*c* ← *c* − *m* × *Q*(*m, c*)
**end if**
**end for**
**until** *C* = ∅
**return** *M*
**end procedure**

**Figure 2:**
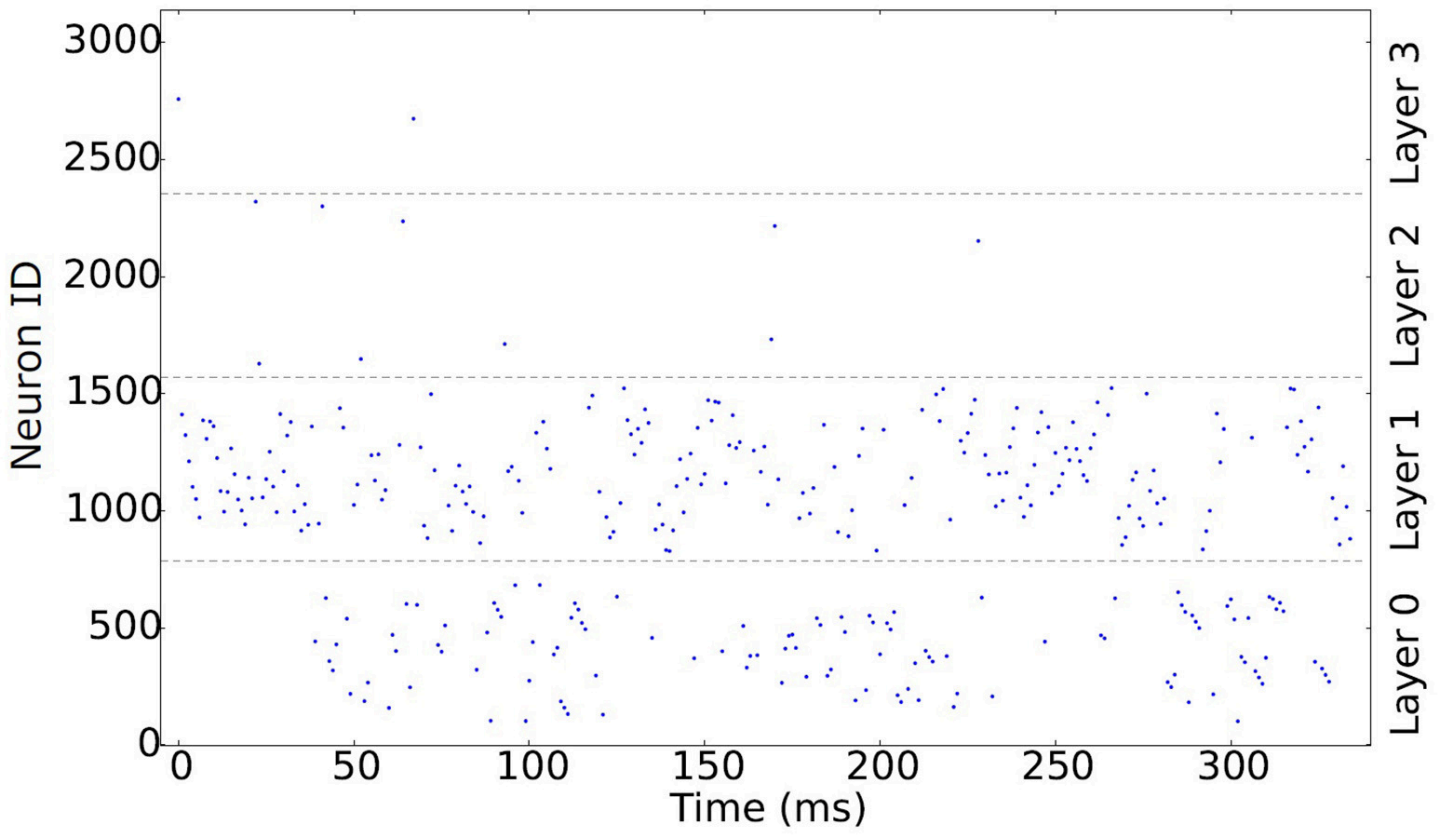
**Raster plot showing the first 30% of the rank-order encoded spikes produced using FoCal at 1 ms intervals**.

After the correction step, the most important information can be recovered using only the first 30% of the spikes (Sen and Furber, 2009). These most significant spikes are shown in Figure 2, which shows the spikes firing at 1 ms intervals. Neurons in Layer 1 emit spikes faster and in larger quantities than any other layer, making it the most important layer. Layers 2 and 3 have few spikes due to the large convolution kernels used to simulate the ganglion cells. One of the main advantages of ROC is that a neuron will only spike once, as can be seen particularly clearly in these two layers. Layers 0 and 1 encode fine detail which can be used to identify what is in the image, while layers 2 and 3 result in blob-like features that should prove useful to location problems.

**Figure 3:**
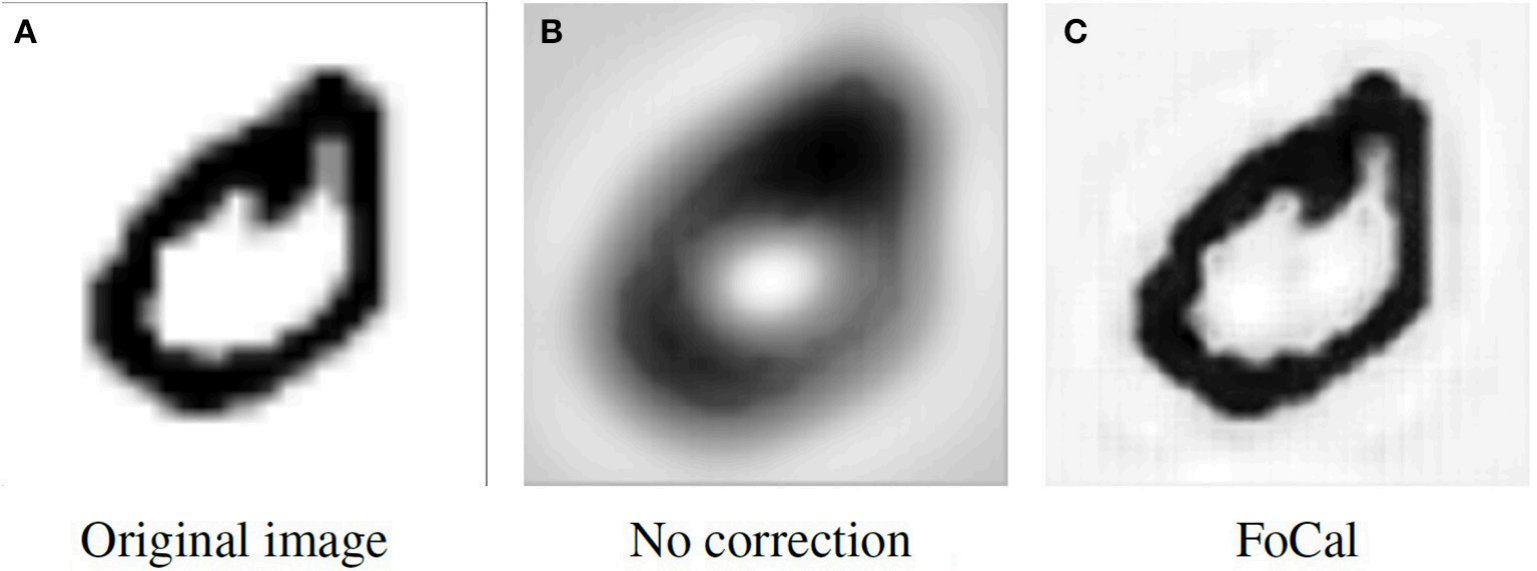
**Reconstruction result comparison. (A)** The original image. **(B)** Reconstruction without overlap correction. **(C)** Reconstruction with overlap correction.

Figure 3 shows the reconstruction results for the two stages of the algorithm. In Figure 3B the reconstruction was applied after the convolution but without the FoCal correction; a blurry image is the result of redundancy in the spike representation. A better reconstruction can be obtained after Algorithm 1 has been applied; the result is shown in Figure 3C.

The source Python scripts to transform images to ROC spike trains, and to convert the results into AER and PyNN spike source arrays, can be found in the dataset website.

#### 2.3.3. DVS sensor output with flashing input

The purpose of including the subset with DVS-recorded flashing digits is to promote research into rapid and accurate “core recognition,” thus to encourage applying non-rate-based algorithms, for example ROC, to short DVS output spike trains.

**Figure 4:**
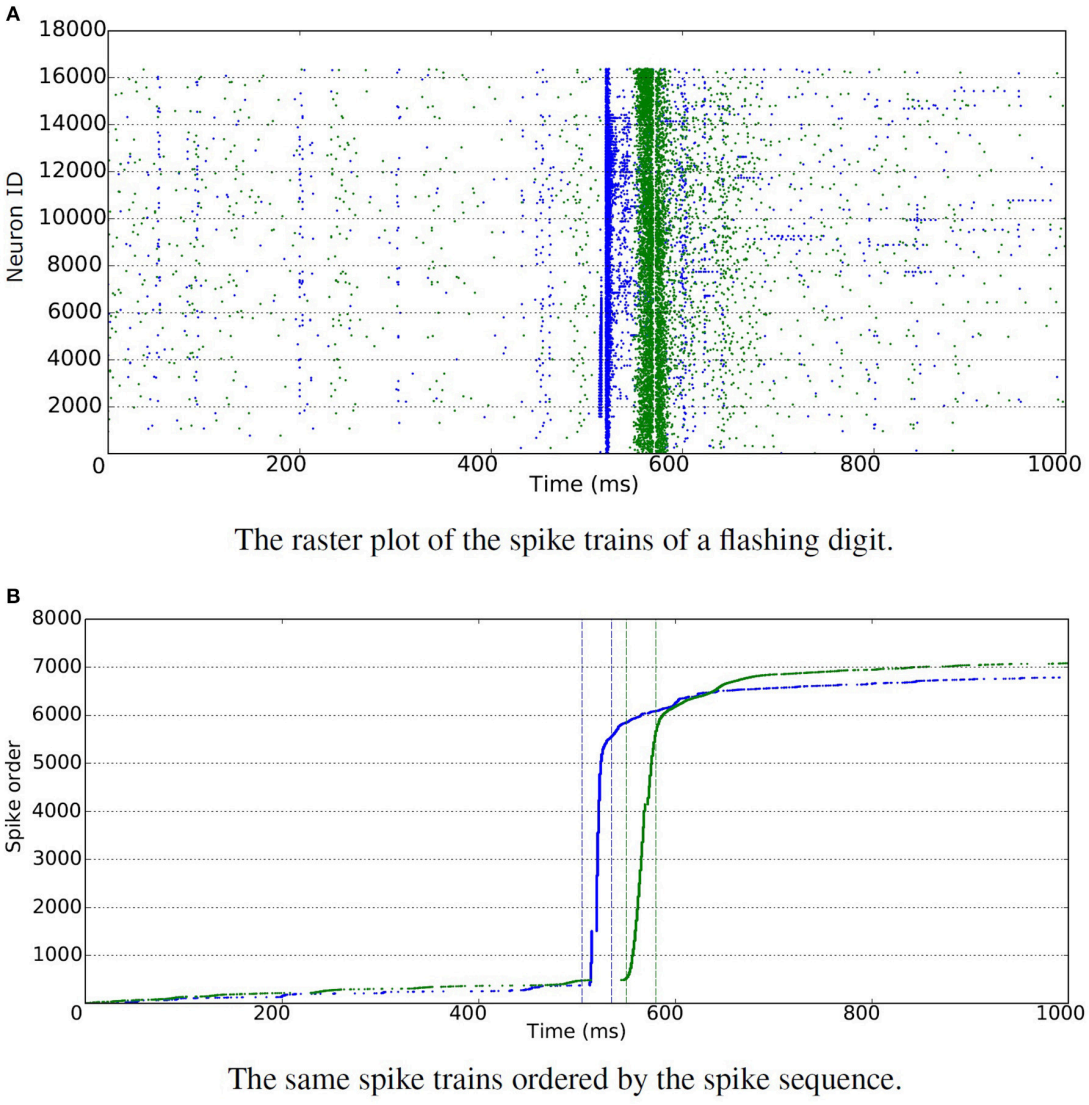
**DVS sensor with flashing input**. Blue is used for “ON” events and green for “OFF” events. **(A)** The raster plot shows spikes generated by individual neurons over time. It is hard to recognize the total number of spikes due to the large number of neurons involved in the figure. Thus all the spikes are ordered in time, and displayed in the figure below. **(B)** The raster plot shows ordered spike sequence over time. The total number of spikes are around 7000 for both “ON” and “OFF” events. The bursty nature of the resulting spikes is illustrated, where most of the spikes occur in a 30 ms time slot.

Each digit was shown alternating with a blank image and each display lasted one second. The digits were displayed on an LCD monitor in front of the DVS retina (Serrano-Gotarredona and Linares-Barranco, 2013) and were placed in the center of the visual field of the camera. Since there are two spike polarities—“ON” indicating an increase in the intensity while “OFF” indicates a decrease—there are “ON” and “OFF” flashing recordings respectively per digit. In Figure 4, the burstiness of the spikes is illustrated where most of the spikes occur in a 30 ms time slot. In total, this subset of the database contains 2 × 60, 000 recordings for training and 2 × 10, 000 for testing.

#### 2.3.4. DVS sensor output with moving input

The subset with DVS recorded moving digits is presented to address the challenges of position- and scale- invariance in computer vision.

MNIST digits were scaled to three different sizes, using smooth interpolation algorithms to increase their size from the original 28 × 28 pixels, and displayed on the monitor with slow motion. The same DVS (Serrano-Gotarredona and Linares-Barranco, 2013) used in Section 2.3.3 captured the movements of the digits and generated spike trains for each pixel in its 128 × 128 resolution. A total of 30, 000 recordings were made: 10 digits, at 3 different scales, 1000 different handwritten samples for each.

### 2.4. Performance evaluation

As a result of the spike-based processing used in SNN models, new concerns about the latency and energy cost arise over performance assessment. Therefore, we propose corresponding evaluation metrics and suggest a sufficient description of SNN models in this section. Once a model is implemented on a neuromorphic platform, the hardware performance can be evaluated by running the particular model. This model-specific assessment provides more robust comparisons between hardware platforms by using the same network topology, neuron and synaptic models, and learning rules. A complementary evaluation methodology is essential to provide common metrics and assess both the model-level and hardware-level performance.

#### 2.4.1. Model-level evaluation

A suggested description of an SNN model is shown in Table 1 where the performance evaluation metrics are in bold and the SNN specific description is in italics.

**Table 1:** **SNN descriptions at the model level**.

**Input**	**Network**	**Training**	**Recognition**
- *converting methods*	- topology	- supervised or not	- classification accuracy
- preprocessing	- *neuron and synaptic type*	- *learning rule*	- *response latency*
		- *biological training time*	- *number of synaptic events*
			- *biological testing time*
			- *input spiking rate*

Because SNNs introduce the time dimension and spike-based processing, additional performance metrics become relevant in addition to classification accuracy: recognition latency and the number of synaptic events. Recognition latency measures how fast spikes are conveyed through the layers of network to trigger the recognition neurons. DiCarlo et al. (2012) considers the rapid (<200 ms) and accurate vision recognition in the brain as the essential problem of object recognition. For real-time systems with live visual inputs, such as robotic systems, a short response latency helps make fast decisions and take rapid action. The latency is measured as the time difference between the first spike generated by the output layer and the first spike from the input layer. A small number of total synaptic events generated by a recognition task indicates the efficiency of the SNN model. A spike event is a synaptic operation evoked when one action potential is transmitted through one synapse (Sharp et al., 2012). Fewer spike events imply lower overall neural activity and lower energy consumption. The number of synaptic events can be measured as “Sopbs,” synaptic operations per biological second.

Alongside the SNN evaluation metrics, a sufficient description of a network model is required so that other researchers can reproduce it and compare it with other models. First of all, the input of an SNN model is specified. The description includes the transformation method for converting raw images to spike trains, and the preprocessing either to images or spikes. Filtering the raw image may ease the classification/recognition task while adding noise may require more robustness in the model. Secondly, as with the evaluation of conventional artificial neural networks, a description of the network characteristics provides the basis for the overall performance evaluation. Sharing the designs not only makes the model reproducible but also inspires fellow scientists to bring new points of view to the problem, generating a positive feedback loop where everybody wins. The main characteristics include the network topology and the neural and synaptic models. The network topology defines the number of neurons used for each layer and the connections between layers and neurons. It is essential to state the types of neural and synaptic model (e.g., current-based LIF neuron) utilized in the network and the parameters configuring them, because neural activities differ significantly between configurations. Any non-neural classifier, sometimes added to aid the design or enhance the output of the network, must also be specified. Thirdly, the training procedure determines the recognition capability of a network model. Specifying the learning algorithm with its mechanism (supervised, semi-supervised and unsupervised) helps the reader understand the core features of the model. A detailed description of new spike-based learning rules will be a great contribution to the field due to the present paucity of spatio-temporal learning algorithms. Most publications reflect the use of adaptations to existing learning rules; details on these modifications should be clear and unambiguous. In conventional computer vision, the number of iterations of training images presented to the network play an important role. Similarly, the biological training time determines the amount of information provided for training an SNN. Finally in the testing phase, as well as the performance evaluation metrics stated above, specific configurations of the input spikes are also essential. This includes details of the way samples are presented to the network: spiking rates, and biological time per test sample. The combination of these two factors determines how much information is presented to the network. Following to the formatted evaluation as in Tables 1, 2 lists a few SNN models of MNIST classification, although some details are missing.

**Table 2:** **Model-level comparison**.

	**Input**	**Network**	**Training**	**Recognition**
Brader et al., 2007	Normalization	Two layer, LIF neurons	Semi-supervised, STDP, calcium LTP/LTD	96.5%
Beyeler et al., 2013	Scaling, V1 (edge), Poisson	V2 (orientation), and competitive decision, Izhikevich neurons	Semi-supervised, STDP, calcium LTP/LTD	91.6% 300 ms per test
Neftci et al., 2013	Thresholding, Synaptic current	Two layer RBM, LIF neurons	Event-driven contrastive divergence (eCD), unsupervised	91.9% 1 s per test
Neftci et al., 2016	Thresholding, Synaptic current	Two layer RBM, LIF neurons	Synaptic Sampling Machine + eCD, unsupervised	95.8% 250 ms per test
Diehl and Cook, 2015	Poisson	Two layers, LIF neurons, inhibitory feedback	Unsupervised, STDP, 200, 000 s per iteration 15 iterations	95%
Diehl et al., 2015	Poisson	ConvNet or Fully connected, LIF neurons	Off-line trained with ReLU, weight normalization	99.1% (ConvNet), 98.6% (Fully connected); 0.5 s per test
Zhao et al., 2015	Thresholding or DVS	Simple (Gabor), Complex (MAX) and Tempotron	Tempotron, supervised	91.3% (Thresholding) 11 s per test 88.1% (DVS), 2 s per test
This paper	Poisson	Four layer RBM, LIF neurons	Off-line trained, unsupervised	94.94% 16 ms latency 1.44M Sopbs
This paper	Poisson	Fully connected decision layer, LIF neurons	K-means clusters, Supervised STDP 18, 000 s of training	92.99% 1 s per test 0.2 s blank 13.82 ms latency 4.17M Sopbs

#### 2.4.2. Hardware-level evaluation

Neuromorphic systems can be categorized as analog, digital, or mixed-mode analog/digital, depending on how neurons, synapses and spike transmission are implemented. Some analog implementations exploit sub-threshold transistor dynamics to emulate neurons and synapses directly in hardware (Indiveri et al., 2011) and are more energy-efficient while requiring less area than their digital counterparts (Joubert et al., 2012). However, the behavior of analog circuits is hard to control through the fabrication process due to transistor mismatch (Linares-Barranco et al., 2003; Pedram and Nazarian, 2006; Indiveri et al., 2011), and achievable wiring densities render direct point-to-point connections impractical for large-scale systems. The majority of mixed-mode analog/digital neuromorphic platforms, such as the High Input Count Analog Neural Network (HI-CANN) (Schemmel et al., 2010), Neurogrid (Benjamin et al., 2014), HiAER-IFAT (Yu et al., 2012), use analog circuits to emulate neurons and digital packet-based technology to communicate spikes as AER events. This enables reconfigurable connectivity patterns, while spike timing is expressed implicitly since typically a spike reaches its destination in less than a millisecond, thus fulfilling the real-time requirement. Digital neuromorphic platforms such as TrueNorth (Merolla et al., 2014) use digital circuits with finite precision to simulate neurons in an event-driven manner to minimize the active power dissipation. Such systems suffer from limited model flexibility, since neurons and synapses are fabricated directly in hardware with only a small subset of parameters under the control of the researcher. The SpiNNaker many-core neuromorphic architecture (Furber et al., 2014) uses low-power programmable cores and scalable event-driven communications hardware allowing neural and synaptic models to be implemented in software. While software modeling provides great flexibility, digital platforms generally have reduced precision (due to the inherent discretisation) and higher energy consumption when compared to analog platforms. Furthermore, the processing cores used in SpiNNaker chips perform better when using integer or fixed-point arithmetic (Hopkins and Furber, 2015). Moreover, the requirement for the models to run in real time leads to constraints on the complexity of model that can be supported.

A direct comparison between neuromorphic platforms is a non-trivial task due to the different hardware implementation technologies as mentioned above. Table 3 attempts to describe the neuromorphic hardware platforms with reference to different aspects of SNN simulation. The scalability of a hardware platform determines the network size limit of a neural application running on it. Considering the various neural and synaptic models, plasticity learning rules and lengths of axonal delays, a programmable platform offers flexibility to support diverse SNNs while a hard-wired system supporting only specific models is advantageous due to its energy-efficiency and simpler design and implementation. The classification accuracy of an SNN running on a hardware system can be different from the software simulation, since hardware implementations may impose limits on the precision used for the membrane potentials of neurons (for the digital platforms) and the synaptic weights. Simulation time is another important measure when running large-scale networks on hardware. Real-time implementation is an essential requirement for robotic systems because of the real-time input from the neuromorphic sensors. Running faster than real time is attractive for large and long simulations. It is interesting to compare the performance of each platform in terms of energy requirements, especially if the platform targets mobile applications and robotics. Some researchers have suggested the use of energy per synaptic event (J/SE) (Sharp et al., 2012; Stromatias et al., 2013) as an energy metric because the large fan in and out of a neuron means that synaptic processing tends to dominate the total energy dissipation during a simulation. Merolla et al. (2014) proposed the number of synaptic operations per second per Watt (Sops/W). These two measures are equivalent, since J/SE × Sops/W = 1.

**Table 3:** **Hardware-level comparison**.

	**System**	**Neuron model**	**Synaptic plasticity**	**Precision**	**Simulation time**	**Energy usage**
SpiNNaker (Stromatias et al., 2013)	Digital, Scalable	Programmable Neuron and Synapse, Axonal delay	Programmable learning rule	11- to 14-bit synapses	Real-time Flexible time resolution	8 nJ/SE
TrueNorth (Merolla et al., 2014)	Digital, Scalable	Fixed models, Config params, Axonal delay	No plasticity	122 bits params and states, 4-bit/ 4 values synapses^a^ (4 signed int + on/off state)	Real-time	26 pJ/SE
Neurogrid (Benjamin et al., 2014)	Mixed-mode, Scalable	Fixed models, Config params	Fixed rule	13-bit shared synapses	Real-time	941 pJ/SE
HI-CANN (Schemmel et al., 2010)	Mixed-mode, Scalable	Fixed models, Config params	Fixed rule	4-bit/ 16 values synapses	Faster than real-time^b^	7.41 nJ/SE(network only)
HiAER-IFAT (Yu et al., 2012)	Mixed-mode, Scalable	Fixed models, Config params	No plasticity	Analog neuron/synapse	Real-time	22-pJ/SE(Park et al., 2014)

a *We consider them 4-bit synapses because it is only possible to choose between 4 different signed integers and whether the synapse is active or not*.

b *A maximum speed-up of up to 10^5^ times real time has been reported*.

However, the typical reported simulation time and energy use for the various platforms is under different SNN models, making the comparisons problematic. Model-specific hardware metrics would provide robust comparisons between platforms and expose how different networks influence the metrics on particular hardware. The proposed evaluation metrics consist of the feasibility, classification accuracy, simulation time, and energy use. A particular SNN model is feasible to run on a particular hardware platform only when the network size is under the platform's limit, the neural and synaptic models are supported, and the learning rule is implemented. CA also plays a role in hardware evaluation because of the precision limits that may be imposed by the platform. Due to the limited hardware resources, simulation time may accelerate or slow down according to the network topology and spike dynamics. Similarly, energy costs vary with different networks and neural and synaptic models.

## 3. Results

In this section, we present two recognition SNN models working on the Poissonian subset of the NE15-MNIST dataset. The network components, training and testing methods are described along the lines set out in Section 2.4.1. The recognition result is evaluated using the proposed metrics: classification accuracy, response latency and number of synaptic events. As tentative benchmarks the models are implemented on SpiNNaker to assess the hardware-level performance against software simulators. Presenting proper benchmarks for vision recognition systems is still under investigation; the case studies only make a first attempt.

### 3.1. Case study I

The first case study is a simple two-layer network where the input neurons receive Poisson spike trains from the dataset and form a fully connected network with the decision neurons. There is at least one decision neuron per digit to classify a test input. The neuron with highest output firing rate classifies a test image as the digit it represents. The model utilizes LIF neurons, and the parameters are all biologically valid, see the listed values in Supplementary Table 2. The LIF neuron model follows the membrane potential dynamics:

(2) $$\begin{array}{l} \tau_{m}\frac{dV}{dt}=V_{rest}-V+R_{m}I_{syn}\left(t\right)\;, \end{array}$$

where τ_*m*_ is the membrane time constant, *V*_*rest*_ is the resting potential, *R*_*m*_ is the membrane resistance and *I*_*syn*_ is the synaptic input current. In PyNN, *R*_*m*_ is presented by *R*_*m*_ = τ_*m*_/*C*_*m*_, where *C*_*m*_ is the membrane capacitance. A spike is generated when the membrane potential goes beyond the threshold, *V*_*thresh*_ and the membrane potential then resets to *V*_*reset*_. In addition, a neuron cannot fire within the refractory period, τ_*refrac*_, after generating a spike.

The connections between the input neurons and the decision neurons are plastic, so the connection weights can be modulated during training with a standard STDP learning rule. The model is described with PyNN and the code is published in the Github repository with the dataset. As a potential benchmark, this system is composed of simple neural models, trained with standard learning rules and written in a standard SNN description language. These characteristics allow the same network to be tested on various simulators, both software- and hardware-based.

Both training and testing use the Poissonian subset of the NE15-MNIST dataset. This makes performance evaluation on different simulators possible with the unified spike source array provided by the dataset. In terms of this case study, the performance of the model was evaluated with both software simulation (on NEST, Gewaltig and Diesmann, 2007) and hardware implementation (on SpiNNaker).

In order to fully assess the performance, different settings were configured on the network, such as network size, input rate and test image duration. For simplicity of describing the system, one standard configuration is set as the example in the following sections.

#### 3.1.1. Training

**Figure 5:**
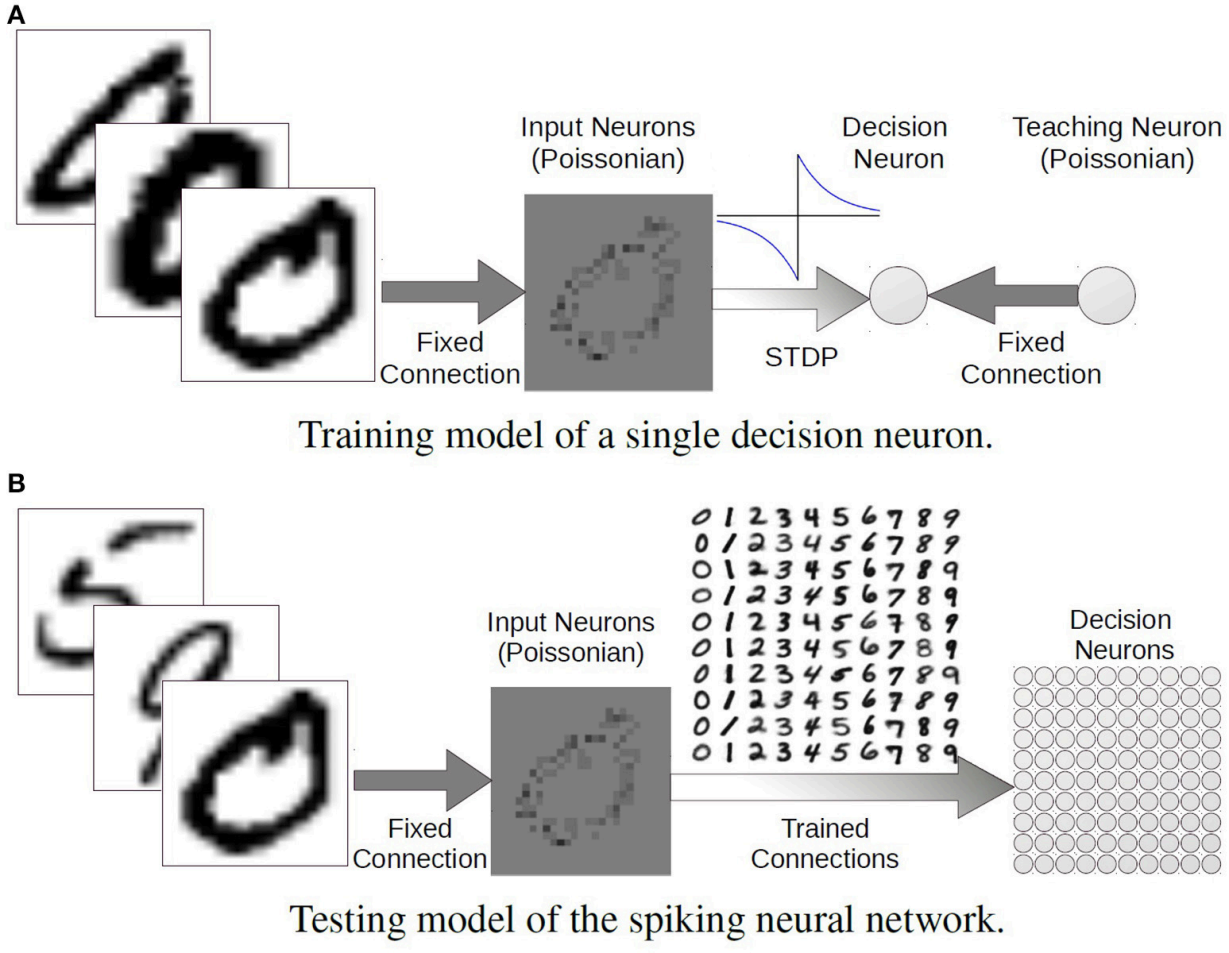
**The (A) training and (B) testing model of the two-layered spiking neural network**.

There are two layers in the model: 28 × 28 input neurons fully connect to 100 decision neurons. Each decision neuron responds to a certain digit template. In the standard configuration, there are 10 decision neurons responding to each digit with slightly different templates. Those templates are embedded in the connection weights between the two layers. Figure 5A shows how the connections to a single decision neuron are tuned.

The training set of 60, 000 hand written digits are firstly classified into 100 classes, 10 subclasses per digit, using K-means clusters. K-means clustering separates a set of data points into K subsets (clusters) according to the Euclidean distance between them. Therefore, each cluster tends to form a boundary within which the data points are near to each other. In this case, all the images of the same digit (a class) are divided into 10 subclasses by assigning *K* = 10. Then the images in a certain subclass are used to train a template embedded in the synaptic weights to the corresponding decision neuron. The firing rates of the input neurons are assigned linearly according to their intensities and the total firing rate of all the 28 × 28 input neurons is normalized to 2000 Hz, that is, the sum of the firing rates of all of the input neurons is 2000 Hz. All the images together are presented for 18, 000 s (about 300 ms per image) during training and at the same time a teaching signal of 50 Hz is conveyed to the decision neuron to trigger STDP learning. The trained weights are plotted in accordance with the positions of the decision neurons in Figure 5B.

#### 3.1.2. Testing

After training the weights of the plastic synapses are set to static, keeping the state of the weights at the last moment of training. However, during training the synaptic plasticity holds a hard limit of 0 on the weight strength, thus excitatory synapses cannot change into inhibitory. To investigate how inhibitory connections influence the classification performance, the weak weights were set to negative with identical strengths. Results show that inhibitory synapses significantly reduced the output firing rates while keeping a good classification ability. Thus, the strategy of replacing weak weights to same negative values was used throughout the case study. The feed-forward testing network is shown in Figure 5B where Poisson spike trains are generated the same way as in the training with a total firing rate of 2000 Hz per image. The input neurons convey the same spike trains to every decision neuron through its responding trained synaptic weights. One test trial contains 10, 000 images in total and each image is presented once and lasts 1 s with a 0.2 s blank period between consecutive images. The output neuron with the highest firing rate determines which digit is recognized. With the standard training configuration, we compared the CA of different simulations of the same SNN model. Using the trained weights from the NEST simulation, the accuracy of the recognition on NEST reached 90.03%, and this accuracy was also achieved on SpiNNaker. When the network was both trained and tested on SpiNNaker the recognition accuracy was 87.41%. Using these weights in NEST yielded a similar result (87.25%). The reduction in CA using the SpiNNaker trained weights was due to precision loss caused by the limited fast memory and the necessity for fixed-point arithmetic to ensure real-time operation. It is inevitable that numerical precision will be below IEEE double precision at various points in the processing chain from synaptic input to membrane potential. The main bottleneck is currently in the ring buffer where the total precision for accumulated spike inputs is 16-bit, meaning that individual spikes are realistically going to be limited to 11- to 14-bit depending upon the probabilistic headroom calculated as necessary from the network configuration and spike throughput (Hopkins and Furber, 2015).

#### 3.1.3. Evaluation

Evaluation starts from the model-level, focusing on the spike-based recognition analysis. As mentioned in Section 2.4.1, CA, response time (latency) and the total number of synaptic events are the main concerns when assessing the recognition performance. In our experiment, two sets of weights were applied: the original STDP trained weights, and scaled-up weights which are 10 times stronger. The spike rates of the test samples were also modified, ranging from 10 to 5000 Hz.

**Figure 6:**
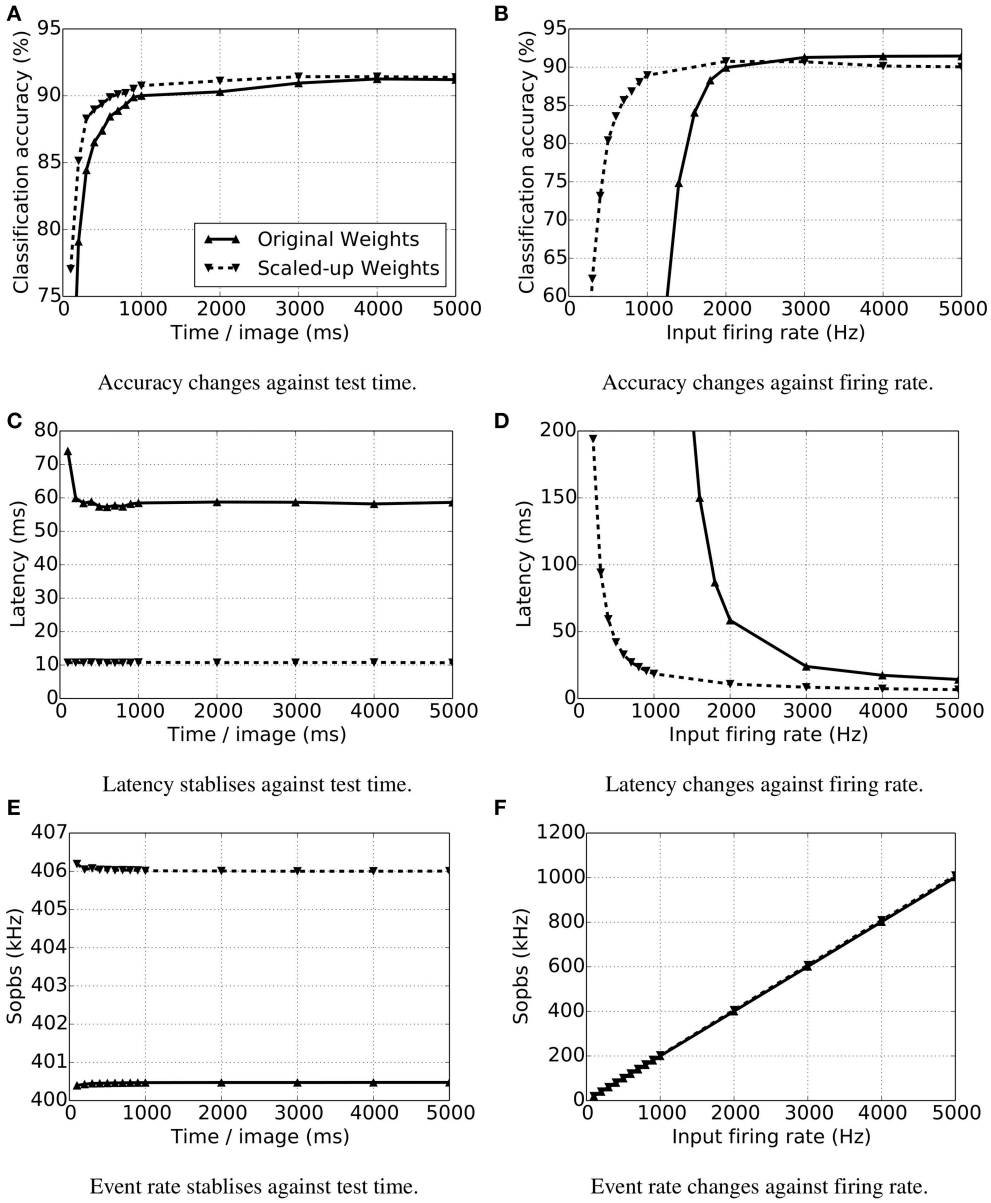
**Accuracy, response time (latency) and synaptic event rate (Sopbs) change over test time and input firing rate per test image**. The test time is the duration of the presence of a single test image, and the input firing rate is the summation of all the input neurons. Original trained weights are used (up-pointing triangles with solid line) as well as the scaled up (× 10) weights (down-pointing triangles with dashed line). **(A)** Accuracy changes against test time, **(B)** accuracy changes firing rate, **(C)** latency stabilizes against test time, **(D)** latency changes against firing rate, **(E)** event rate stabilizes against test time, **(F)** event rate changes against firing rate.

We found that accuracy depends largely on the time each sample is exposed to the network and the sample spike rate (Figure 6). Figure 6A shows that the CA is better as exposure time increases. The longer an image is presented, the more information is gathered by the network, so the accuracy climbs. Classification accuracy also increases when input spike rates are augmented (Figure 6B). Given that the spike trains injected into the network are more intense, the decision neurons become more active, and so does the output disparity between them. Nonetheless, it is important to know that these increases in CA have a limit, as is shown in the aforementioned figures. With stronger weights, the accuracy is much higher when the input firing rate is less than 2000 Hz.

The latency of an SNN model is the result of the input firing rates and the synaptic weights. We measured the latency of each test by getting the time difference of the first spike generated by any decision neuron in the output layer and the first spike of the input layer. As the input firing rates grow, there are more spikes arriving at the decision neurons, triggering them to spike sooner. A similar idea applies to the influence of synaptic weights. If stronger weights are taken, then the membrane potential of a neuron reaches its threshold earlier. Figure 6D indicates that the latency is shortened with increasing input firing rates with both the original and scaled-up weights. When the spiking rate is less than 2000 Hz, the network with stronger weights has a much shorter latency. As long as there are enough spikes to trigger the decision neurons to spike, increasing the test time will not make the network respond sooner (Figure 6C).

At the default configuration of the SNN model, each input neuron connects to all of the 100 decision (output) neurons with both excitatory and inhibitory projections. Thus, the synaptic events happening in the inter-layer connections are 200 times the total input firing rate. Figure 6E shows the stable Sopbs of the entire network when the input firing rate is held at 2000 Hz and the test time increases. The firing rates of the output layer are relatively small, and are 0.1% and 1.5% of the total Sopbs using original and scaled-up weights respectively. The variations in the total Sopbs lie in the firing rate of the output layers only, and the stronger connections lead to the higher firing rates. Likewise, the output neurons are more active with stronger connection weights, and the gap widens as the input firing rate increases, see Figure 6F. Although the variations in the Sopbs climbs to around 8 kHz, it is not obvious in the figure because the output firing rates are relatively low and therefore so are the differences.

The network size not only influences the accuracy of a model but also the time taken for simulation on specific platforms, thus impacting the energy usage on the hardware. For the purpose of comparing the accuracy, simulation time, number of synaptic events and energy usage, different configurations have been tested on NEST (working on a PC with CPU: i5-4570 and 8G memory) and on SpiNNaker. The same experiment was run 4 times with different random seeds; the average performance estimation is listed in Table 4. The input rates in all of the tests are 5000 Hz, and each image is presented for 1 s with a 0.2 s blank period between consecutive images during which the model receives no input. The configurations only differ in the number of templates (subclasses/clusters) per digit.

**Table 4:** **Comparisons of NEST (N) on a PC and SpiNNaker (S) performance averaged over 10 trials**.

**Subclasses per digit**		**1**	**10**	**50**	**100**	**1000**
Avg. response latency (ms)		18.03	14.25	13.82	13.57	13.15
Avg. synaptic events (Sopbs)		83,691.48	835,274.98	4,173,392.03	8,343,559.69	83,385,785.67
Accuracy	N	79.63 ±0.23	91.42 ±0.13	92.99 ±0.15	87.05 ±0.21	89.63 ±0.08
(%)	S	79.57 ±0.31	91.39 ±0.09	92.99 ±0.08	87.00 ±0.26	89.58 ±0.24
Avg. SIM	N	445.09	503.21	767.67	1131.09	12, 027.75
time (s)	S			12,000		
Power	N	20	20	20	19	17
(W) Energy	N	8.90	10.06	15.34	21.50	208.25
(KJ)	S	4.56	4.56	4.92	5.28	18.00

**Figure 7:**
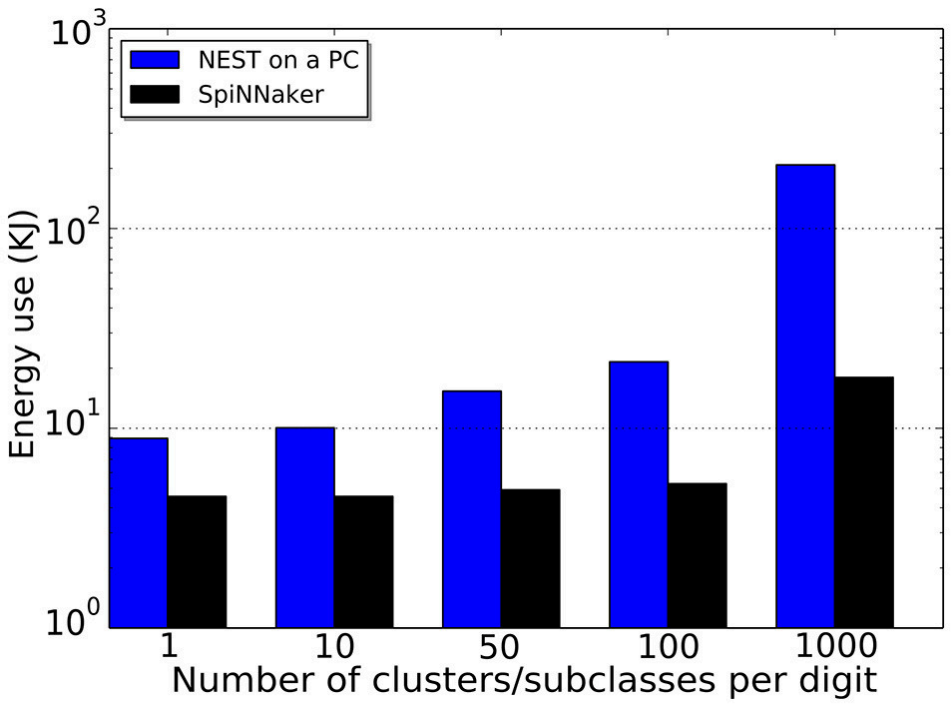
**Energy usages of different network size both using NEST (blue) on a PC and SpiNNaker (black)**.

As the network size grows there are more decision neurons and synapses connecting to them, thus the simulation time on NEST increases. On the other hand, SpiNNaker works in (biologically) real time and the simulation time becomes shorter than the NEST simulation when 1000 patterns per digit (1000 decision neurons per digit) are used. The NEST simulation was run on a desktop PC, and the power use was measured by a meter socket and estimated by subtracting the usage of idle OS operation from the usage running the simulation. In doing so, the power consumption of the resources needed to run the simulation is better approximated. The SpiNNaker test was run on a Spin4 board which has 48 chips and exposed pins to measure electrical quantities. A built-in Arduino board provided a measurement read out of the power usage of the chips. For the same goal of estimating just the required resources, only the active chips were measured. Even with the smallest network, SpiNNaker wins in the energy cost comparison, see Figure 7. Among different network configurations, the model with 500 decision neurons (50 clusters per digit) reaches the highest recognition rate of 92.99% on average having a latency of 13.82 ms mean and 2.96 ms standard deviation. And there are standard deviations of 2.57% on CA and of 1.17 ms on the latency over 10 testing digits. The total number of synaptic events is around 4.17M Sopbs, where only 7K spikes are generated in the output layer. The NEST simulation costs 767.67 s on average for the entire 12, 000 s biological-time test, 20 W in power use on the PC and 15.35 KJ of energy, while SpiNNaker works in real time using 4.92 KJ of energy at a power of 0.41 W (see Table 4). This result provides a baseline for comparison with other SNN models and neuromorphic hardwares, and no optimization is applied.

### 3.2. Case study II

This section aims to review and reinterpret results from previously published studies (Stromatias et al., 2015a,b,c), which utilized the identical off-line trained^2^ spiking DBN as presented by O'Connor et al. (2013).

Deep learning architectures and, in particular, Convolutional Networks (LeCun et al., 1998) and Deep Belief Networks (DBNs) (Hinton et al., 2006) have been characterized as one of the breakthrough technologies of the decade (Hof, 2013). One of the advantages of these type of network is that their performance can be increased by adding more layers (Hinton et al., 2006).

However, state-of-the-art deep networks comprise a large number of layers, neurons and connections resulting in high energy demands, communication overheads, and high response latencies. This is a problem for mobile and robotic platforms which may have limited computational and power resources but require fast system responses.

O'Connor et al. (2013) proposed a method to map off-line trained DBNs into a spiking neural network and take advantage of the real-time performance and energy efficiency of neuromorphic platforms. This led initially to an implementation on an event-driven Field-Programmable Gate Array (FPGA) called Minitaur (Neil and Liu, 2014) and then on the SpiNNaker platform (Stromatias et al., 2015b). This particular DBN comprises 784 neurons for the input layer, two hidden layers with 500 neurons each, and an output layer with 10 neurons. This is abbreviated as a 784-500-500-10 architecture. Simulations take place on a software spiking neural network simulator, Brian (Goodman and Brette, 2008), and results are verified on the SpiNNaker platform.

#### 3.2.1. Training

DBNs consist of stacked Restricted Boltzmann Machines (RBMs), which are fully connected recurrent networks but without any connections between neurons in the same layer. Training is performed unsupervised using the standard Contrastive Divergence (CD) rule (Hinton et al., 2006) and only the output layer is trained in a supervised manner. The main difference between spiking DBNs and traditional DBNs is the activation function used for the neurons. O'Connor et al. (2013) proposed the use of the Siegert approximation (Jug et al., 2012) as the activation function, which returns the expected firing rate of an LIF neuron (Equation 2) given the input firing rates, the input weights, and standard neuron parameters. Further details regarding the training process can be found in O'Connor et al. (2013).

#### 3.2.2. Testing

After the training process the learnt synaptic weights can be used in a spiking neural network which consists of LIF neurons with delta-current synapses. Supplementary Table 3 shows the LIF parameters used in the simulations. These parameters were chosen by O'Connor et al. (2013) to train this spiking DBN network. Using the same network and parameters allowed us to have a direct comparison between the power requirements and numerical precision, for different software and hardware platforms (Matlab, Brian, Minitaur, SpiNNaker).

The pixels of each MNIST digit from the testing set are converted into Poisson spike trains as described in Section 2.3.1. The CA was chosen as the performance metric of the spiking DBN, which is the percentage of the correctly classified digits over the whole MNIST testing set.

#### 3.2.3. Evaluation

Neuromorphic platforms may have limited hardware resources to store the synaptic weights (Schemmel et al., 2010; Merolla et al., 2014). In order to investigate how the precision of the weights affects the CA of a spiking DBN the double-precision floating-point weights of the offline-trained network were converted to various fixed-point representations. The following notation will be used throughout this paper, Q*m.f*, where *m* signifies the number of bits for the integer part (including the sign bit) and *f* the number of bits used for the fractional part.

**Figure 8:**
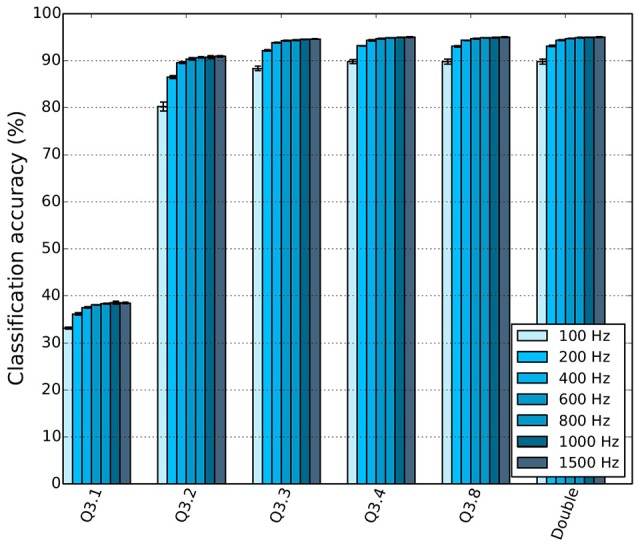
**DBN classification accuracy (CA) as a function of the weight bit precision for different input firing rates (Stromatias et al., 2015c)**.

Figure 8 shows the effect of reduced weight bit precision on the CA for different input firing rates on the Brian simulator. Using the same weight precision of Q3.8, SpiNNaker achieved a CA of 94.94% when 1500 Hz was used for the input population (Stromatias et al., 2015b). With the same firing rates and weight precision, Brian achieved a CA of 94.955%. Results are summarized in Table 5. The slightly lower CA of the SpiNNaker simulation indicates that not only the weight precision but also the precision of the membrane potential affects the overall classification performance. Stromatias et al. (2015c) showed that spiking DBNs are capable of maintaining a high CA even for weight precisions down to Q3.3, while they are also remarkably robust to high levels of input noise regardless of the weight precision.

**Table 5:** **Classification accuracy (CA) of the same DBN running on different platforms**.

**Simulator**	**CA (%)**	**Weight precision**
Matlab	96.06	Double floating point
Brian	95.00	Double floating point
Brian	94.955	Q3.8
SpiNNaker	94.94	Q3.8

**Figure 9:**
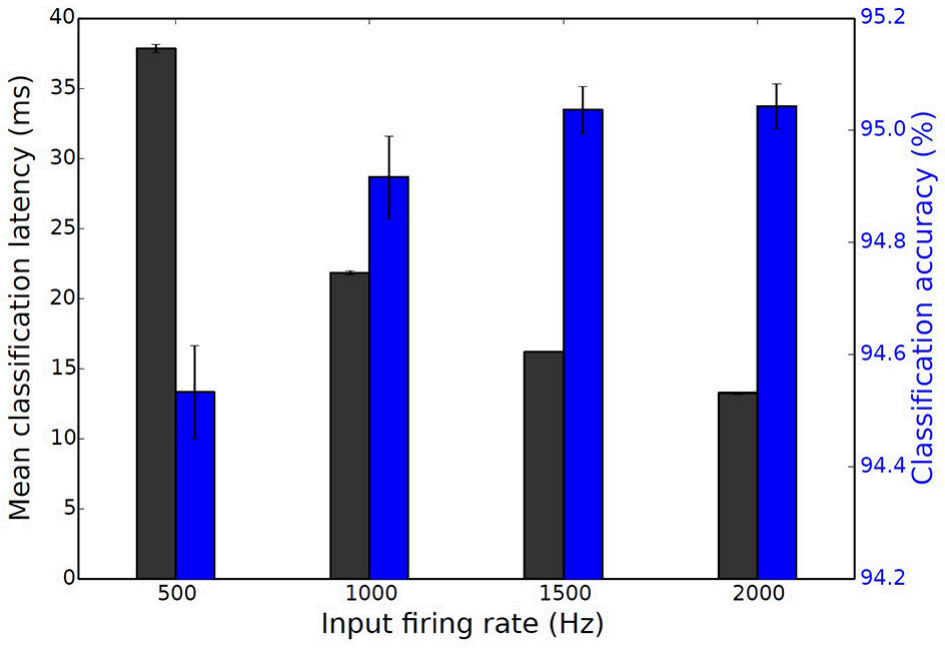
**Mean classification latency (black) and classification accuracy (blue) as a function of the input firing rate for the spiking DBN**. Results are averaged over 4 trials, error bars show standard deviations (Stromatias et al., 2015b).

A similar experiment to the one presented for Case Study I was performed; its purpose was to establish the relation that input spike rates hold with latency and classification accuracy. The input rates were varied from 500 Hz to 2000 Hz and the results are summarized in Figure 9. Simulations ran in Brian for all 10, 000 MNIST digits of the testing set and for 4 trials. Supplementary Figure 2 shows a histogram of the classification latencies on SpiNNaker when the input rates are 1500 Hz. The mean classification latency for the particular spiking DBN on SpiNNaker is 16 ms which is identical to the Brian simulation seen in Figure 9.

**Figure 10:**
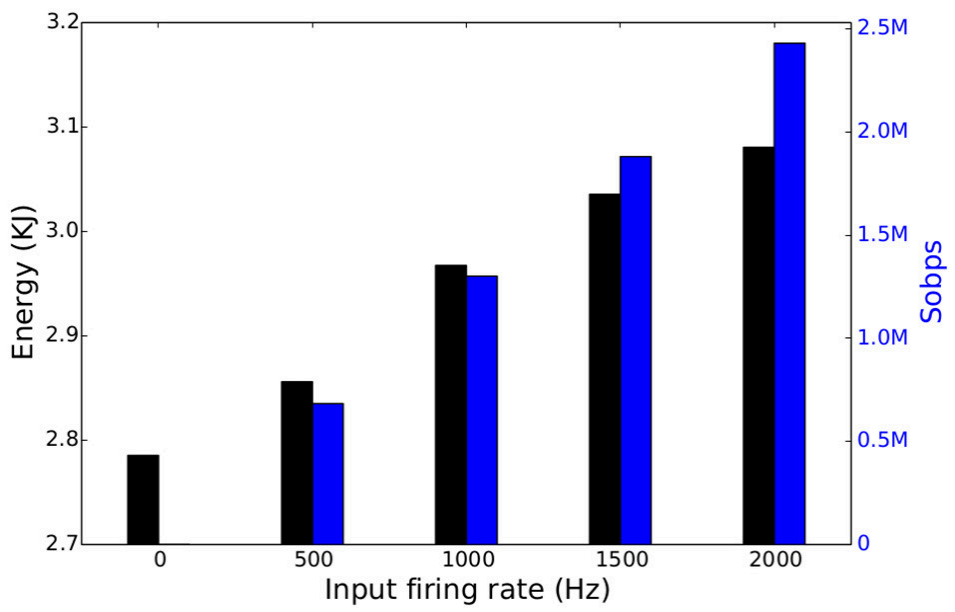
**Total energy consumption (black) and number of SE per second (blue) of a spiking DBN running on a single SpiNNaker chip as a function of the total input firing rate**.

Finally, this particular spiking DBN ran on a single SpiNNaker chip (16 ARM9 cores) and dissipated about 0.3 W when 1, 500 spikes per second per digit were used. The number of generated synaptic events was 1.88M Sopbs and less than 2.97 KJ of energy was consumed running the whole testing set over 10, 000 s, as seen in Figure 10. The identical network executed on Minitaur (Neil and Liu, 2014), an event-driven FPGA implementation, dissipated 1.5 W when 1000 spikes per image were used, and achieved a CA of 92.0%.

## 4. Discussion

### 4.1. Summary of the work

This paper puts forward the NE dataset as a baseline for comparisons of vision based SNN models and neuromorphic platforms. It contains spike-based versions of existing widely-used databases in the vision recognition field. Since new problems will continue to arise before vision becomes a solved question, the dataset will evolve as research progresses. The conversion methods for transforming images and videos into spike trains will advance. The number of vision datasets will increase and the corresponding evaluation methodologies will evolve. The dataset aims to provide unified spike-based vision benchmarks and complementary evaluation methodologies to assess the performance of SNN algorithms.

The first version of the dataset is published as NE15-MNIST, which contains four different spike representations of the MNIST stationary hand-written digit database. The Poissonian subset is intended for benchmarking existing rate-based recognition methods. The rank-order coded subset, FoCal, encourages research into spatio-temporal algorithms on recognition applications using only small numbers of input spikes. Fast recognition can be verified on the DVS recorded flashing input subset, since just 30 ms of useful spike trains are recorded for each image. Looking forward, the continuous spike trains captured from the DVS recorded moving input can be used to test mobile neuromorphic robots. Orchard et al. (2015) have presented a neuromorphic dataset using a similar approach, but the spike trains were obtained with micro-saccades. This dataset aims to convert static images to neuromorphic vision input, while the recordings of moving input in our paper are intended to promote position-invariant recognition. Therefore, the datasets complement each other.

The proposed complementary evaluation methodology is essential to assess both the model-level and hardware-level performance of SNNs. In addition to classification accuracy, response latency and the number of synaptic events are specific evaluation metrics for spike-based processing. Moreover, it is important to describe an SNN model in sufficient detail to share the network design, and relevant SNN characteristics were highlighted in the paper. The network size of an SNN model that can be built on a hardware platform will be constrained by the scalability of the hardware. Neural and synaptic models are limited to the ones that are physically implemented, unless the hardware platform supports programmability. Any attempt to implement an on-line learning algorithm on neuromorphic hardware must be backed by synaptic plasticity support. Therefore, running an identical SNN model on different neuromorphic hardware exposes the capabilities of such platforms. If the model runs smoothly on a hardware platform, it then can be used to benchmark the performance of the hardware simulator in terms of simulation time and energy usage. Classification accuracy (CA) is also a useful metric for hardware evaluation because of the limited precision of the membrane potential and synaptic weights.

This dataset makes the comparison of SNNs with conventional recognition methods possible by using converted spike representations of the same vision databases. As far as we know, this is the first attempt at benchmarking neuromorphic vision recognition by providing public a spike-based dataset and evaluation metrics. In accordance with the suggestions from Tan et al. (2015), the evaluation metrics highlight the strengths of spike-based vision tasks and the dataset design also promotes the research into rapid and low energy recognition (e.g., flashing digits). Two benchmark systems were evaluated using the Poissonian subset of the NE15-MNIST dataset. These example benchmarking systems demonstrated a recommended way of using the dataset, describing the SNN models and evaluating the system performance. The case studies provide baselines for robust comparisons between SNN models and their hardware implementations.

### 4.2. The future direction of an evolving database

The database will be expanded by converting more popular vision datasets to spike representations. As mentioned in Section 1, face recognition has become a hot topic in SNN approaches, however there is no unified spike-based dataset to benchmark these networks. Thus, the next development step for our dataset is to include face recognition databases. While viewing an image, saccades direct high-acuity visual analysis to a particular object or a region of interest and useful information is gathered during the fixation of several saccades in a second. It is possible to measure the scan path or trajectory of the eyeball and those trajectories show particular interest in eyes, nose and mouth while viewing a human face (Yarbus, 1967). Therefore, our plan is also to embed modulated trajectory information to direct the recording using DVS sensors to simulate human saccades.

There will be more methods and algorithms for converting images to spikes. Although Poisson spikes are the most commonly used external input to an SNN system, there are several *in-vivo* recordings in different cortical areas showing that the inter-spike intervals (ISI) are not Poissonian (Deger et al., 2012). Thus Deger et al. (2012) proposed new algorithms to generate superposition spike trains of Poisson processes with dead-time (PPD) and of Gamma processes. Including novel spike generation algorithms in the dataset is one aspect of future work which will be carried out.

Each encounter of an object on the retina is unique, because of the illumination (lighting condition), position (projection location on the retina), scale (distance and size), pose (viewing angle), and clutter (visual context) variabilities. But the brain recognizes a huge number of objects rapidly and effortlessly even in cluttered and natural scenes. In order to explore invariant object recognition, the dataset will include the NORB (NYU Object Recognition Benchmark) dataset (LeCun et al., 2004), which contains images of objects that are first photographed in ideal conditions and then moved and placed in front of natural scene images.

Action recognition will be the first problem of video processing to be introduced in the dataset. The initial plan is to use the DVS retina to convert the KTH and Weizmann benchmarks to spiking versions. Meanwhile, providing a software DVS retina simulator to transform frames into spike trains is also on the schedule. By doing this, a huge number of videos, such as those in YouTube, can automatically be converted into spikes, therefore providing researchers with more time to work on their own applications.

Overall, it is impossible for the dataset proposers to provide enough datasets, converting methods and benchmarking results, thus we encourage other researchers to contribute to the dataset. Researchers can contribute their data to the dataset, allowing future comparisons using the same data source. They can also share their spike conversion algorithms by generating datasets to promote the corresponding recognition methods. Neuromorphic hardware owners are welcome to provide benchmarking results to compare their system's performance.

## Author contributions

QL, the main author, contributing to the dataset, performance evaluation and case study I. GP, contributes to the dataset and performance evaluation, and paper writing. ES, contributes to the performance evaluation and case study II. TS, contributes to the dataset and discussions on performance evaluation. SF, takes part in discussions and reviewing the paper.

### Conflict of interest statement

The authors declare that the research was conducted in the absence of any commercial or financial relationships that could be construed as a potential conflict of interest.

## Footnotes

^1^http://yann.lecun.com/exdb/mnist/

^2^https://github.com/dannyneil/edbn/

## Supplementary material

Supplementary Table 1 **Simulation parameters for FoCal ganglion cells**.

Supplementary Table 2 **Parameter setting for the current-based LIF neurons using PyNN**.

Supplementary Table 3 **Default parameters of the Leaky Integrate-and-Fire Model used in the DBN simulations**.

Supplementary Figure 1 **Results of convolving the image spikes with the simulated ganglion cell layers using the FoCal algorithm before correcting for filter overlap. (A)** The original image. **(B–E)** The result of convolving the image with the layer 1 (smallest) OFF-center to layer 4 (largest) ON-center kernels respectively.

Supplementary Figure 2 **Histogram of the classification latencies for the MNIST digits of the testing set when the input rates are set to 1500 Hz**. The mean classification latency of the spiking DBN on SpiNNaker is 16 ms (Stromatias et al., 2015b).
