# Supplementary figures and images for: Benchmarking Spike-Based Visual Recognition: A Dataset and Evaluation
Source: Front Neurosci. 2016 Nov 2;10:496. doi: 10.3389/fnins.2016.00496 (PMC5090001; doi:10.3389/fnins.2016.00496)

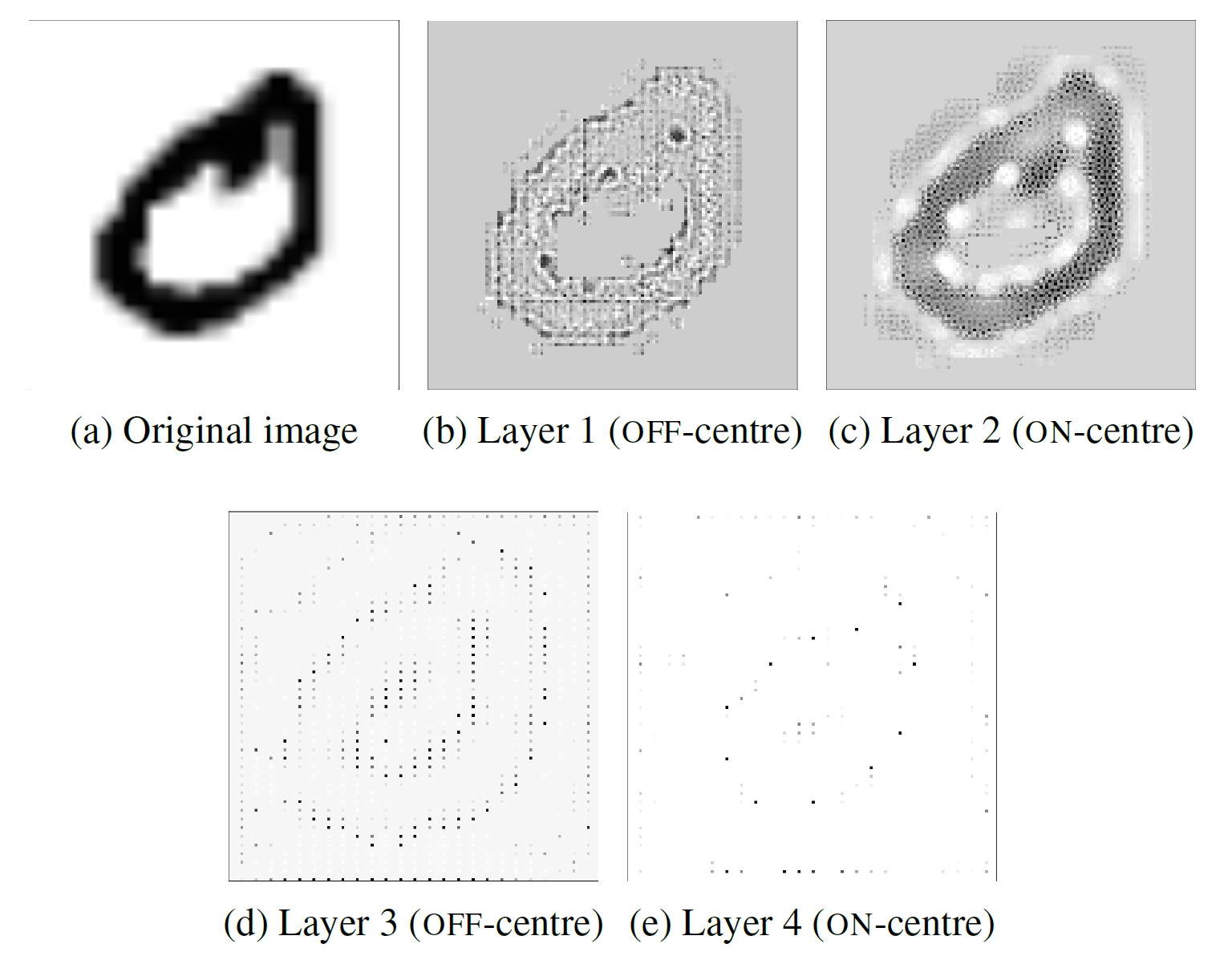

Supplement: Supplementary Figure 1 — Results of convolving the image spikes with the simulated ganglion cell layers using the FoCal algorithm before correcting for filter overlap. (A) The original image. (B–E) The result of convolving the image with the layer 1 (smallest) OFF-center to layer 4 (largest) ON-center kernels respectively. [file Image1.jpg]

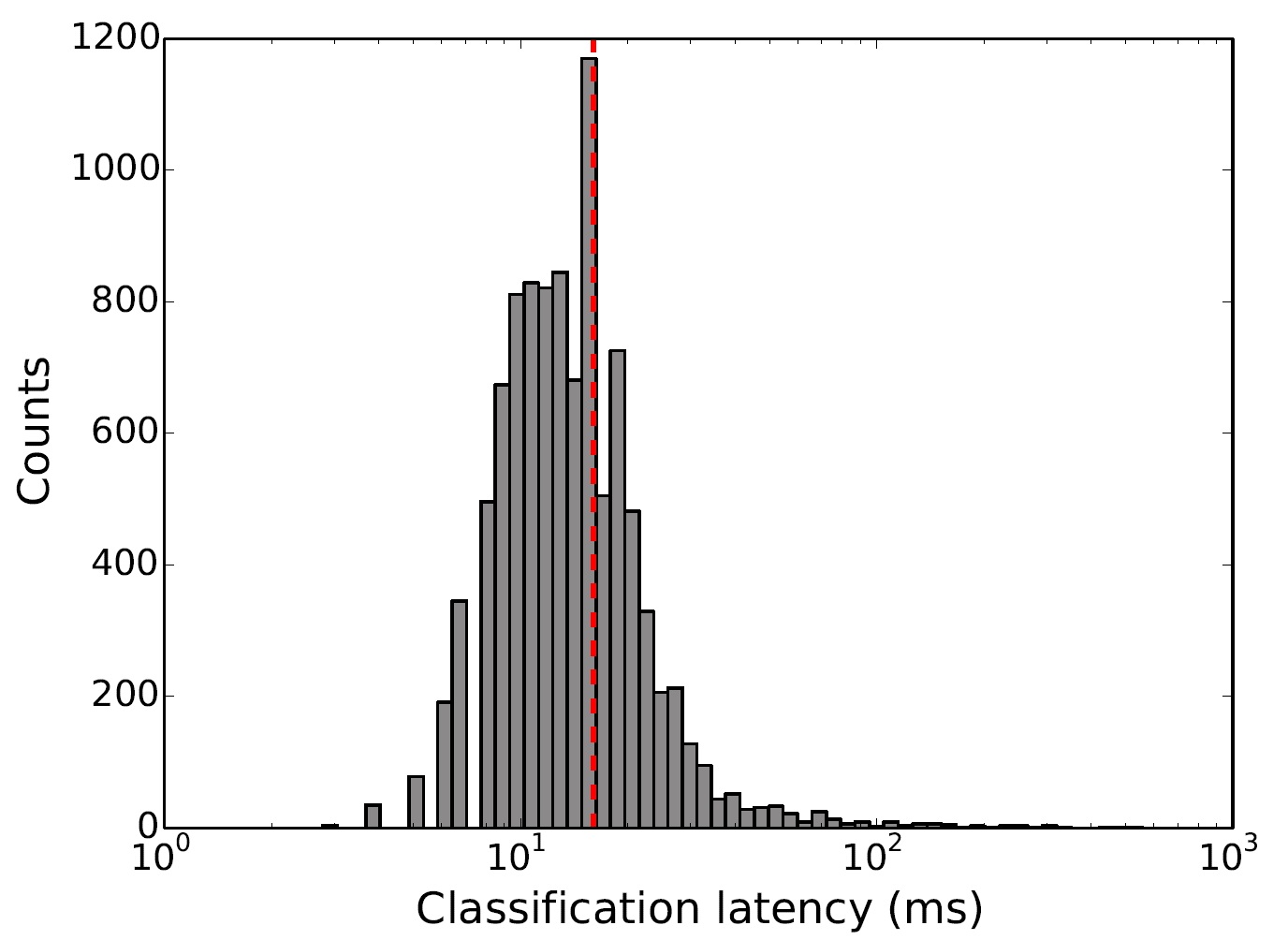

Supplement: Supplementary Figure 2 — Histogram of the classification latencies for the MNIST digits of the testing set when the input rates are set to 1500 Hz. The mean classification latency of the spiking DBN on SpiNNaker is 16 ms (Stromatias et al., 2015b). [file Image2.jpg]
